# Potato Spindle Tuber Viroid RNA-Templated Transcription: Factors and Regulation

**DOI:** 10.3390/v10090503

**Published:** 2018-09-17

**Authors:** Shachinthaka D. Dissanayaka Mudiyanselage, Jie Qu, Nancy Tian, Jian Jiang, Ying Wang

**Affiliations:** 1 Department of Biological Sciences, Mississippi State University, Starkville, MS 39762, USA; sdd292@msstate.edu (S.D.D.M.); jiang@biology.msstate.edu (J.J.); 2 Department of Pathology and Laboratory Medicine, University of Cincinnati, Cincinnati, OH 45237, USA; quje@ucmail.uc.edu; 3 College of Medicine, Ohio State University, Columbus, OH 43210, USA; nancy.tian@osumc.edu

**Keywords:** viroid replication, hepatitis delta virus, TFIIIA, RPL5, Pol II, alternative splicing, HDAg

## Abstract

Viroids are circular noncoding RNAs that infect plants. Without encoding any protein, these noncoding RNAs contain the necessary genetic information for propagation in hosts. Nuclear-replicating viroids employ DNA-dependent RNA polymerase II (Pol II) for replication, a process that makes a DNA-dependent enzyme recognize RNA templates. Recently, a splicing variant of transcription factor IIIA (TFIIIA-7ZF) was identified as essential for Pol II to replicate potato spindle tuber viroid (PSTVd). The expression of TFIIIA-7ZF, particularly the splicing event, is regulated by a ribosomal protein (RPL5). PSTVd modulates its expression through a direct interaction with RPL5 resulting in optimized expression of TFIIIA-7ZF. This review summarizes the recent discoveries of host factors and regulatory mechanisms underlying PSTVd-templated transcription processes and raises new questions that may help future exploration in this direction. In addition, it briefly compares the machinery and the regulatory mechanism for PSTVd with the replication/transcription system of human hepatitis delta virus.

## 1. Introduction

Viroids are single-stranded circular noncoding RNAs. Without protein-coding capacity, viroids utilize plant machinery for replication and spread, often leading to disease [1,2]. There are two families of viroids known to date, *Avsunviroidae* and *Pospiviroidae*. The *Avsunviroidae* contains four members that replicate in chloroplasts and have branched terminal domains [1,2,3]. Although some evidence implied the involvement of plastid-encoded RNA polymerase (PEP) in the replication of viroids in the *Avsunviroidae* [4], compelling evidence, based on the sensitivity of avocado sunblotch viroid replication to a specific inhibitor targetoxin, supports a nucleus-encoded RNA polymerase (NEP) as the replication enzyme [5]. All other viroids belong to the *Pospiviroidae* family. In general, viroids in the *Pospiviroidae* replicate in the nucleus, relying on the activity of DNA-dependent RNA polymerase II (Pol II) [1,2]. The intrinsic RNA-dependent RNA polymerase (RdRP) activity of DNA-dependent RNA polymerases (DdRP) was first reported by Dezélée et al. in 1974 [6]. However, the identification of Pol II as the authentic polymerase for the replication of potato spindle tuber viroid (PSTVd) [7], after the assumption regarding involvement of a tomato RdRP [8], was the first to demonstrate the intrinsic RdRP activity of a DdRP in a natural biological process. This discovery was strongly supported by the evidence that (1) purified Pol II can transcribe PSTVd template and generate full-length (−)-strand product and (2) α-amanitin, at a specific concentration against Pol II activity, inhibited PSTVd-templated transcription [7]. Following this discovery, similar observations confirmed the involvement of Pol II in the replication of various viroids in the *Pospiviroidae* based on α-amanitin treatment and/or Pol II binding [9,10,11,12,13,14,15,16,17,18,19,20]. The evidence showing Pol II binding to circular PSTVd RNA template in vivo has also been reported recently [21].

## 2. The Discovery and Biogenesis of TFIIIA in Plants

Pol II is a multi-subunit enzyme that requires orchestrated actions of various co-factors to ensure successful transcription [22,23]. Since the discovery of PSTVd-templated transcription by Pol II, what factors are involved in this process has been an outstanding question. With the discovery of the recurring loop E RNA motif on both PSTVd and 5S rRNA, 5S rRNA binding proteins, such as transcription factor IIIA (TFIIIA) and ribosomal protein L5 (RPL5), have been speculated to be host factors for PSTVd [24]. TFIIIA is a single copy gene deeply conserved in eukaryotes [25]. In mammalian cells and yeast, TFIIIA is the required transcription factor for the transcription of 5S rRNA, and transports 5S rRNA to the cytoplasm after transcription [26]. RPL5 binds to 5S rRNA and transports it to nucleoli for ribosomal assembly [26]. Since TFIIIA is a transcription factor, it aroused great interest to test whether TFIIIA is involved in PSTVd-templated transcription by Pol II.

TFIIIA has a characteristic organization of nine C2H2 type zinc finger domains (ZFs) [25,27]. Despite the conserved structure, the primary nucleotide sequences vary greatly among species, posing difficulties in identifying orthologues. The first plant TFIIIA gene from *Arabidopsis thaliana* was reported in 2003 [28], followed by two reports showing that the TFIIIA gene in land plants has a conserved alternative splicing pattern [29,30]. One splicing variant encodes the canonical TFIIIA with nine ZFs (TFIIIA-9ZF), while the other retains a short 5S rRNA-mimic intron introducing a premature stop codon (PTC) after the sequence corresponding to the first two ZFs. We named the latter variant as *TFIIIA-7ZF* [21] because it contains a large open reading frame (ORF) encoding seven ZFs nearly identical to the C-terminus of TFIIIA-9ZF in addition to the mini upstream ORF (uORF) containing those two ZFs. It is noteworthy that the uORF is translatable in an in vitro assay [29].

The biogenesis of TFIIIA-7ZF is under debate. The presence of the PTC near the 5′ end of *TFIIIA-7ZF* led to the assumption that this variant is regulated by the non-sense mediated decay (NMD) pathway [29,30,31]. However, a recent study showed that transcripts regulated by NMD usually have a very long 3′ untranslated region in plants [32]. This may not be the case for *TFIIIA-7ZF* because it contains a normal ORF after the PTC. By contrast, the uORF encoding two ZFs may play a regulatory role in translating the downstream ORF (TFIIIA-7ZF), as exemplified in several recent studies in plants [33,34,35,36,37]. Early work failed to demonstrate the translation of TFIIIA-7ZF [29,30]. However, with the development of TFIIIA-specific antibodies, immune-blotting results from *A. thaliana* and *Nicotiana benthamiana* clearly showed the presence of TFIIIA-7ZF protein in plants [21,31]. Layat et al. [31] concluded that the production of TFIIIA-7ZF is a result of proteolytic process. This conclusion was based entirely on complementing the potential lethality of a homozygous *A. thaliana* mutant (SALK_008626C) that has a T-DNA insertion in TFIIIA locus. However, the T-DNA insertion is at the end of C-terminal region leaving all the seven ZFs unaffected and the homozygous plant is viable according to *Arabidopsis* biological resource center (https://www.arabidopsis.org/servlets/SeedSearcher?action=detail&stock_number=SALK_008626c). Therefore, we tend to believe that TFIIIA-7ZF is directly translated from *TFIIIA-7ZF*. This assumption is supported by (1) the molecular weight of observed TFIIIA-7ZF protein in line with the predicted ORF [21,31] and (2) the altered expression of the splicing regulator (RPL5) directly leading to changes in the TFIIIA-7ZF accumulation (see below for more details) [38].

## 3. TFIIIA-7ZF Is Required for PSTVd-Templated Transcription by Pol II

After the discovery of plant TFIIIA gene, efforts have been made to test whether TFIIIA is the host factor operating in PSTVd-templated transcription by Pol II. Eiras et al. [39] first showed that TFIIIA-9ZF cloned from *Arabidopsis* can bind to (+)-PSTVd in vitro. Following this report, Wang et al. [21] further confirmed that both TFIIIA-9ZF and TFIIIA-7ZF can bind to (+)-PSTVd in vitro and in vivo. TFIIIA-9ZF has a much higher affinity with (+)-PSTVd as compared with that of TFIIIA-7ZF [21,39]; however, TFIIIA-7ZF, but not TFIIIA-9ZF, interacts with (−)-PSTVd in vitro and in vivo, mimicking the binding capacity of Pol II [21]. The major binding site for TFIIIA-7ZF was mapped to the lower portion of PSTVd left terminal domain [21], coinciding with the loop structures critical for replication in vivo [40] and Pol II binding [41], as well as near the transcription initiation site [12]. In contrast, TFIIIA-9ZF binds to the right terminal domain [20], where RNA motifs critical for trafficking reside [40,42]. Interestingly, TFIIIA-9ZF exclusively accumulated in nucleoli in PSTVd-infected leaves, while TFIIIA-7ZF located in both nucleoplasm and nucleoli [21]. The difference in spatial distribution patterns makes TFIIIA-7ZF, but not TFIIIA-9ZF, more likely to function together with Pol II, an enzyme localized in the nucleoplasm. Manipulating the expression of TFIIIA-7ZF affected PSTVd titers in the nucleus, which further supported that TFIIIA-7ZF is the bona fide transcription factor for PSTVd. The most direct evidence was from an in vitro transcription assay that resulted in the production of longer-than-unit-length (−)-PSTVd. This assay was successful by using purified TFIIIA-7ZF, but not purified bovine serum albumin or TFIIIA-9ZF, to mix with purified Pol II and circular PSTVd template [21]. Therefore, TFIIIA-7ZF is a dedicated factor critical for Pol II-dependent transcription on PSTVd RNA template to generate longer-than-unit-length products. However, it remains to be determined if TFIIIA-7ZF is also critical for transcription initiation.

Another remaining question is the functional implication of the interaction between TFIIIA-9ZF and PSTVd. TFIIIA-9ZF is often the dominant form in plants and binds to PSTVd with a higher affinity [21,39]. Due to technical challenges, TFIIIA-9ZF loss-of-function assay was not achieved, although over-expression of TFIIIA-9ZF did not appear to affect PSTVd replication [21]. It is interesting to note that the TFIIIA-9ZF binding site partially overlaps with that of VirP1, another PSTVd host factor [21,42,43]. Studies have shown that VirP1 binds to RY motifs close to PSTVd right terminal domain and regulates PSTVd replication [43,44]. A recent report showed that VirP1 can facilitate the nuclear import of a viral satellite RNA, which raises a possibility that VirP1 may also regulate the nuclear import of PSTVd [45]. In this regard, it is worth testing whether VirP1 and/or TFIIIA-9ZF regulate PSTVd nuclear import. On the other hand, it is also of interest to understand the critical ZFs in TFIIIA-7ZF for RNA-templated transcription, which will shed light on the molecular mechanism in regulating the RdRP activity of a DdRP and the coordination of multiple ZFs in function.

## 4. Other Factors Involved in PSTVd-Templated Transcription Catalyzed by Pol II

**Figure 1:**
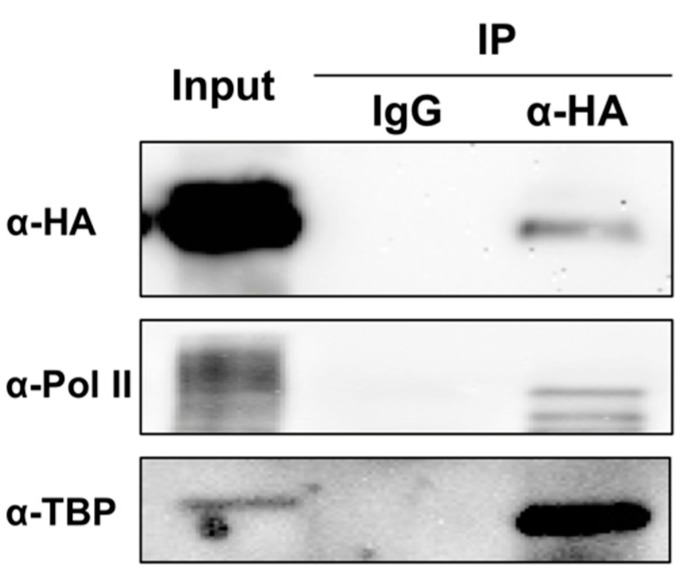
Co-immunoprecipitation of TFIIIA-7ZF/Pol II/TBP. Overexpressed and HA-tagged TFIIIA-7ZF served as a bait and successfully pulled down endogenous Pol II largest subunit and TBP. IgG- and α-HA-conjugated resins were used as negative and positive treatments, respectively. The experimental methods were described previously [21]. IP, immunoprecipated fractions.

Transcription is a complex process that requires a group of factors functioning cooperatively with a polymerase. The composition of the required machinery is clear for DNA-dependent transcription through decades of studies [22,23]. By contrast, the required factors involved in RNA-templated transcription by Pol II or other DdRPs remain elusive, despite that Pol II utilizes the same active site for both RNA and DNA templates [46]. Studies on human hepatitis delta virus (HDV; relying on Pol II for transcription and replication) demonstrated the formation of a preinitiation complex that is composed of multiple general transcription factors, such as TFIIA, TFIIB, TFIID (TATA-box binding protein; TBP), TFIIE, TFIIF, TFIIH, and TFIIS [47]. This observation shows that transcription machinery for DNA-templates and RNA-templates share certain similarities. However, a hepatitis delta antigen (HDAg-S) is required in the Pol II elongation process [48], indicating the presence of unique factors for RNA-templated transcription by DdRPs. Based on our unpublished data and numerous studies on TFIIIA-9ZF truncated protein [49,50,51,52], TFIIIA-7ZF does not possess 5S rDNA-binding capacity. The RNA-binding capacity allows TFIIIA-7ZF to act as an RNA-templated transcription factor, which is required for the production of longer-than-unit-length PSTVd by Pol II [21]. Besides TFIIIA-7ZF and the largest and second largest subunits of Pol II, other factors for PSTVd-templated transcription await identification. In an effort to show the in vivo interaction of TFIIIA-7ZF and Pol II using co-immunoprecipitation, we detected the enrichment of TBP in the immunoprecipitated fraction using TFIIIA-7ZF as a bait (Figure 1). This result supports the involvement of TBP in the machinery for PSTVd-templated transcription in plants, but future functional analyses are needed. In addition, it will be insightful to understand the protein composition of purified Pol II used for PSTVd-templated in vitro transcription.

## 5. RPL5 as a Regulator of *TFIIIA* Splicing and PSTVd Replication

RPL5 is a well-studied ribosomal protein that directly binds to 5S rRNA to form 5S RNP, which traffics to nucleoli for ribosomal assembly. There is no distinct RNA-binding domain in RPL5, and instead, nearly the whole protein is required for 5S rRNA binding [26]. A recent study found that alternative splicing of *TFIIIA* is regulated by RPL5. RPL5 binds to the 5S rRNA-mimic intron in *TFIIIA* pre-mRNA thus facilitating the intron removal [30]. PSTVd directly interacts with RPL5 through PSTVd central conserved region (CCR) [38]. The RPL5-PSTVd interaction is abolished when the loop E motif in CCR is disrupted. Upon PSTVd infection, the expression of RPL5 is induced about 2-fold. However, this induction fails to repress TFIIIA-7ZF accumulation, likely due to reduced protein availability through a direct RPL5-PSTVd interaction in vivo [38]. This observation suggested that PSTVd impairs RPL5 regulation over TFIIIA splicing during infection. Further over-expression of RPL5 in infected plants repressed TFIIIA-7ZF expression and reduced PSTVd titer [38]. Therefore, RPL5 as a negative factor for PSTVd, may be exploited in future breeding efforts. Furthermore, RPL5 and TFIIIA-7ZF constitute an RPL5/TFIIIA-7ZF regulatory cascade critical for PSTVd replication.

## 6. Possible PSTVd RNA Conformations during Transcription

**Figure 2:**
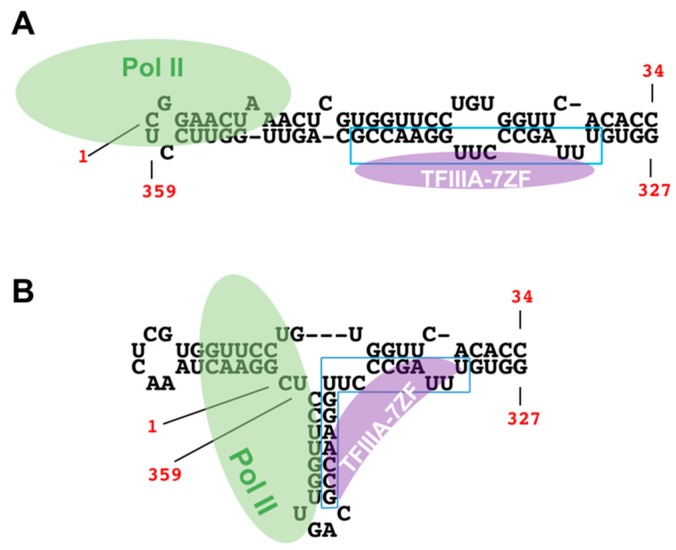
Two possible left terminal conformations during replication. (**A**) Traditional rod-shape left terminal conformation; (**B**) Alternative bifurcated left terminal conformation. The mapped TFIIIA-7ZF binding region is highlighted in a blue box.

PSTVd and members in the *Pospiviroidae* fold into a rod-shaped structure with five domains: the left and right terminal domains; the pathogenicity domain; the central domain; and, the variable domain [53]. This structural organization has been supported by various studies using a combination of in vivo and in vitro approaches [54,55,56]. Pol II transcription on (+)-strand PSTVd RNA initiates at the left terminal domain, according to the mapped initiation site [12] and the electron microscopy images [13]. Purified Pol II was also shown to bind with a PSTVd fragment containing the left terminal domain [41]. Therefore, it is intuitive to assume that PSTVd stays in a rod-shaped conformation during transcription and the transcription initiates at the nucleotide 1 in the left terminal loop (loop 1). However, in this view half of the Pol II enzyme might be outside of the template at the initiation step (Figure 2A) and Pol II might be forced to make one or more “U turns” on a rod-shaped template during elongation. Alternatively, a bifurcated conformation might form when interacting with TFIIIA-7ZF and Pol II during transcription initiation (Figure 2B). This bifurcated conformation has been determined by several studies and probably exists in various nuclear-replicating viroids, despite that these viroids have distinct primary sequences [55,57,58]. It is noteworthy that the wildtype PSTVd fragment binding with Pol II in an in situ assay [41] has an intrinsic capacity to fold into a bifurcated conformation or a rod-shaped conformation. The bifurcated terminal structure might provide a structural support for the Pol II docking at the initiation step and also might favor the activity of Pol II via a stem-loop structure [46]. Furthermore, it is not against a recent structural analysis of PSTVd genomic RNA in vivo [54], because this alternative structure might transiently exist in only a few molecules that are masked by the dominant rod-shaped conformation. It is important to note that studying PSTVd RNA conformations during transcription processes (i.e., initiation, elongation, etc.) is critical for understanding PSTVd biogenesis and broadly for RNA-templated transcription by DdRPs. Given the lack of conclusive experimental evidence for either of the models, it is worth testing which conformation is involved in the transcription initiation in the near future.

**Figure 3:**
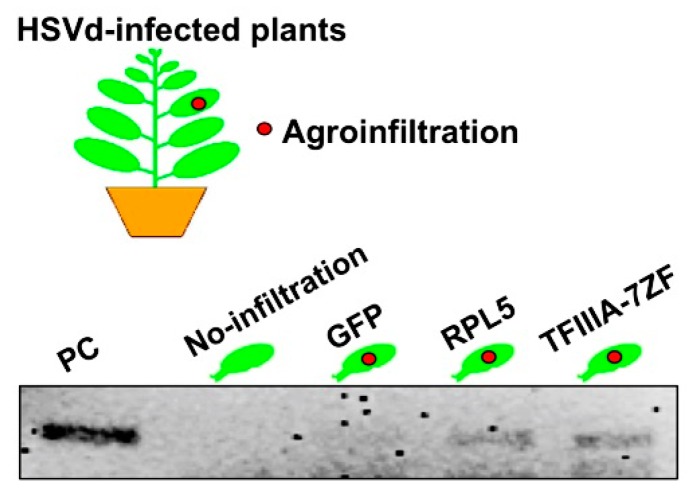
RNA-immunoprecipitation supporting that TFIIIA-7ZF and RPL5 bind to HSVd in vivo. HA-tagged proteins (GFP, RPL5, and TFIIIA-7ZF) were expressed in HSVd-infected *N. benthamiana* plants via agroinfiltration and served as baits. RPL5 and TFIIIA-7ZF as baits successfully pulled down HSVd RNA. No-infiltrated and GFP-expressing leaves served as negative controls. The experimental methods were described previously [21]. PC, monomeric HSVd cDNA served as a size marker.

PSTVd and 10 other viroids in the genus *Pospiviroid* share conserved CCRs in terms of sequences and structures [56]. Thus, it is reasonable to assume that these viroids all exploit the RPL5/TFIIIA-7ZF regulatory cascade for replication. However, other members in the *Pospiviroidae* possess CCRs with various sequences and structures [56]. Whether these viroids also employ the RPL5/TFIIIA-7ZF regulatory cascade for replication remains an outstanding question. In an in vivo RNA immunoprecipitation assay, we found that TFIIIA-7ZF and RPL5 cloned from *N. benthamiana* also bind to hop stunt viroid (HSVd) (Figure 3), which has a distinct CCR as compared with PSTVd [56,59]. How RPL5 recognizes various CCRs needs to be determined. In addition, it is also worth testing whether the sequence variations in both RPL5 and TFIIIA-7ZF from various hosts contribute to PSTVd host ranges. Lastly, this system offers a platform to test if the RPL5/TFIIIA-7ZF regulatory cascade is employed for replication by other viroids in the family *Pospiviroidae*.

**Figure 4:**
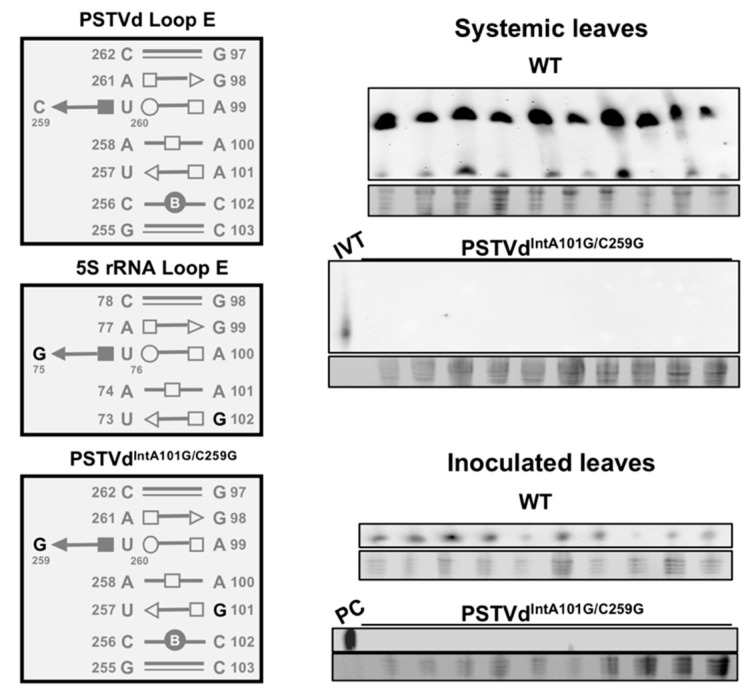
Loss of infectivity of a potato spindle tuber viroid (PSTVd) variant with 5S rRNA loop E sequences. Left panel displays loop E structural arrangements in wildtype (WT) PSTVd, *N. benthamiana* 5S rRNA, and a loop E swapped PSTVd^IntA101G/C259G^. The right panel shows northern blots detecting the infection of WT PSTVd and PSTVd^IntA101G/C259G^ in local and systemic leaves of *N. benthamiana* plants. PC, a verified RNA sample from PSTVd-infected leaves served as a positive control. IVT, the unit-length in vitro transcript served as a control. Note: only circular PSTVd (an indicator of replication) is shown in the northern blots for inoculated leaves. The “○, ⎕, ∆” symbols depict Watson-Crick, Hoogsteen, and sugar edges, respectively. The hollow and solid symbols represent *trans* and *cis* glycosidic orientations, respectively. These symbolic annotations of loop E 3-dimensional structures were previously explained in detail [63,65].

Comparison of TFIIIA-7ZF/TFIIIA-9ZF and RPL5 binding with 5S rRNA and PSTVd generates interesting insights. It is well understood that TFIIIA-9ZF binds to the 5S rRNA region covering helixes I, III, and V as well as loops A, B, and E [60,61]. By contrast, RPL5 binds to helix II and loop C of 5S rRNA [62]. Albeit PSTVd and 5S rRNA share the recurring loop E motif [63,64], TFIIIA variants do not bind to PSTVd loop E but recognize loops in two terminal domains [21]. Instead, RPL5 binds to PSTVd loop E [38]. Replacing PSTVd loop E with the sequences of *N. benthamiana* 5S rRNA loop E results in a non-infectious strain (Figure 4), despite that both loop E motifs likely share similar 3-dimensional structural arrangements [64]. These observations raise the question regarding what additional mechanisms determine the specificity in protein-RNA interactions other than RNA motifs.

## 7. Transcription on (−)-Strand PSTVd

It is generally accepted that transcription from (+)-strand genome to (−)-intermediates of nuclear-replicating viroids are catalyzed by Pol II [7,21]. However, it is less clear whether the transcription from (−)-PSTVd intermediates to multimeric (+)-PSTVd intermediates is also Pol II-dependent. Studies using different inhibitors led to different conclusions [14,18]. More importantly, the assays in these studies mixed both (−)- and (+)-PSTVd that can be templates for each other, generating difficulties in data interpretation. It is notable that both TFIIIA-7ZF and Pol II, but not TFIIIA-9ZF, bind to (−)-PSTVd in vitro and in vivo [21], rendering them possible to function in the transcription on the (−)-PSTVd template. Nevertheless, it awaits further tests for clarification.

## 8. RNA-Templated Transcription of PSTVd and HDV: A Brief Comparison

**Figure 5:**
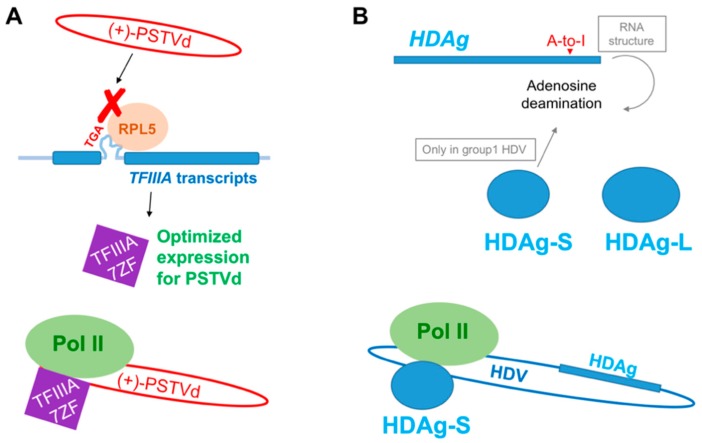
Possible regulatory mechanisms for the expression of TFIIIA-7ZF and HDAg-S. (**A**) PSTVd directly binds to RPL5 to regulate alternative splicing of *TFIIIA*, resulting in optimized TFIIIA-7ZF expression and PSTVd replication; (**B**) The expression of HDAg-S and HDAg-L is regulated through A-to-I RNA editing at a specific site. Hepatitis delta virus (HDV) RNA structure may affect the editing. In addition, HDAg-S itself may also impact the editing efficiency in group 1 HDV.

Emerging evidence outlines plant machinery for PSTVd-templated transcription with a composition of Pol II’s largest and second largest subunits, TFIIIA-7ZF [7,21], and potentially TBP. TFIIIA-7ZF is a splicing variant from a single copy gene, TFIIIA. The alternative splicing process of TFIIIA is regulated by RPL5 through binding to the 5S rRNA-mimic intron. RPL5 binding facilitates the intron removal and favors the production of TFIIIA-9ZF [30]. PSTVd modulates its own replication through a direct interaction with RPL5, resulting in impaired RPL5 regulation over *TFIIIA* splicing and optimized expression of TFIIIA-7ZF (Figure 5A) [38]. It is interesting to compare this mechanism with the transcription on HDV RNA template. HDV-templated transcription relies on the orchestrated activities of Pol II [66] and the virus-encoded HDAg-S [48]. Recently, a study showed the formation of a preinitiation complex on the HDV RNA template [47]. HDV contains only one HDAg gene that encodes two proteins (HDAg-S and HDAg-L) with distinct roles. HDAg-L functions in viral assembly [67] while the shorter form HDAg-S is required for replication and transcription of HDV by Pol II [48,66,68]. Unlike the alternative splicing mechanism that regulates the expression of two TFIIIA proteins, there is a specific A-to-I RNA editing resulting in a translational read-through. This A-to-I RNA editing is catalyzed by the RNA adenosine deaminase (ADAR1). The exact regulatory mechanism for this editing is under debate. Currently, it is believed that group 1 HDV viruses, but not other sub-types, can use HDAg-S to influence the RNA editing [69]. Besides, it has also been shown that HDV RNA secondary structures and viral replication may influence this RNA editing (Figure 5B) [70].

## 9. Summary

Some DdRPs possess RdRP activity, reflecting their likely evolution from RdRPs that first arose to transcribe RNA templates [46]. Recent progress has identified TFIIIA-7ZF as a dedicated transcription factor for Pol II-dependent transcription using PSTVd RNA template. The expression of TFIIIA-7ZF is regulated by the RPL5-based splicing mechanism, constituting an RPL5/TFIIIA-7ZF regulatory cascade. PSTVd likely interferes with this regulatory cascade through a direct interaction with RPL5, resulting in efficient replication. These findings also raise critical questions for future research, revolving around (1) the utilization of the RPL5/TFIIIA-7ZF regulatory cascade by other nuclear-replicating viroids or not; (2) the required components for Pol II-centered transcription machinery; (3) the PSTVd structural conformation during Pol II-dependent transcription; and, (4) the determination of substrate specificity of TFIIIA-7ZF and RPL5.
